# Letter from the Editor-in-Chief

**DOI:** 10.19102/icrm.2017.081105

**Published:** 2017-11-15

**Authors:** Moussa Mansour


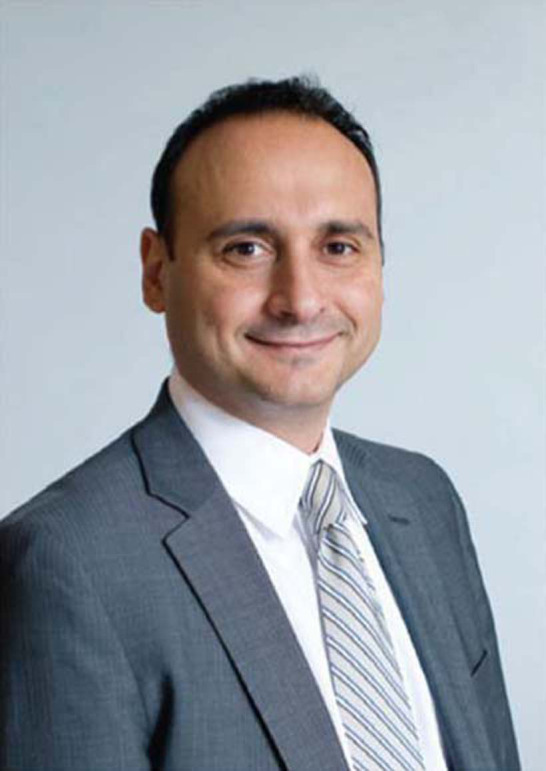


Dear Readers,

Though original research is often interesting and enjoyable in itself to read, we as clinicians must also strive to remain up to date on the mechanisms, pathophysiology, and relevant ongoing clinical trials of different diseases that may affect our patients. This issue of *The Journal of Innovations in Cardiac Rhythm Management* contains a very informative manuscript titled “When Silence Isn’t Golden: The Case of 'Silent' Atrial Fibrillation.” In it, Dr. James Reiffel reviews the prevalence of silent atrial fibrillation (AF) and the risk of thromboembolism associated with it. Furthermore, he discusses available methods of monitoring for AF, including ambulatory and implantable loop recorders. Published landmark studies such as EMBRACE, CRYSTAL AF, PREDATE AF, ASSERT-II, REVEAL AF are reviewed in detail, and ongoing studies such as LOOP and GRAF-AF are also described, giving readers a look at the current state of clinical trials on the management of silent AF.

There has been significant interest in the topic of silent AF within the last five years. In 2012, the ASSERT study showed that the subclinical atrial tachyarrhythmias occurred in approximately 10% of patients^1^ within a few months of either pacemaker or defibrillator implantation, respectively. Moreover, the study demonstrated that these patients had a significant risk of stroke and systemic embolism. Since that time, many medical device and consumer product companies have been striving to develop advanced tools for the detection of silent AF, and many of the resulting items have become commercially available.

The science of detecting AF is rapidly progressing, as evidenced by the numerous research initiatives being completed. However, the pace of the effort in conducting studies investigating the management of silent AF has been slower. This has resulted in challenging situations for cardiologists and health care providers, who must utilize the findings of monitoring tools in the management of their patients but without the robust science to guide such. Specifically, there remains no consensus on the burden, frequency, and duration of silent AF that necessitates treatment. Thus, it is of utmost importance that large, well-designed clinical studies are initiated soon so as to help answer this unmet clinical need.

I hope that you enjoy reading this issue of *The Journal of Innovations in Cardiac Rhythm Management*.

Best wishes for a happy Thanksgiving.

Sincerely,


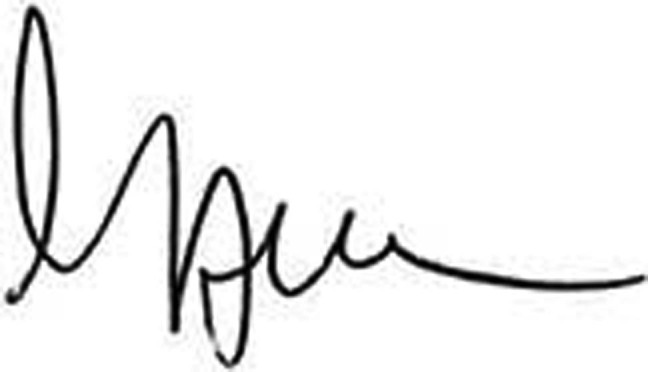


Moussa Mansour, MD, FHRS, FACC

Editor-in-Chief

The Journal of Innovations in Cardiac Rhythm Management

MMansour@InnovationsInCRM.com

Director, Atrial Fibrillation Program

Jeremy Ruskin and Dan Starks Endowed Chair in Cardiology

Massachusetts General Hospital

Boston, MA 02114
